# Assessing informative tract segmentation and nTMS for pre-operative planning

**DOI:** 10.1016/j.jneumeth.2023.109933

**Published:** 2023-08-01

**Authors:** Oeslle Lucena, Jose Pedro Lavrador, Hassna Irzan, Carla Semedo, Pedro Borges, Francesco Vergani, Alejandro Granados, Rachel Sparks, Keyoumars Ashkan, Sebastien Ourselin

**Affiliations:** a King’s College London, London, UK; b King’s College Hospital Foundation Trust, London, UK

**Keywords:** Navigated Transcranial Magnetic Stimulation (nTMS), Deep Learning, Uncertainty Quantification, Tractography, Tract Segmentation

## Abstract

**Background:**

Deep learning-based (DL) methods are the best-performing methods for white matter tract segmentation in anatomically healthy subjects. However, tract annotations are variable or absent in clinical data and manual annotations are especially difficult in patients with tumors where normal anatomy may be distorted. Direct cortical and subcortical stimulation is the gold standard ground truth to determine the cortical and sub-cortical lo- cation of motor-eloquent areas intra-operatively. Nonetheless, this technique is invasive, prolongs the surgical procedure, and may cause patient fatigue. Navigated Transcranial Magnetic Stimulation (nTMS) has a well-established correlation to direct cortical stimulation for motor mapping and the added advantage of being able to be acquired pre-operatively.

**New method:**

In this work, we evaluate the feasibility of using nTMS motor responses as a method to assess corticospinal tract (CST) binary masks and estimated uncertainty generated by a DL-based tract segmentation in patients with diffuse gliomas.

**Results:**

Our results show CST binary masks have a high overlap coefficient (OC) with nTMS response masks. A strong negative correlation is found between estimated uncertainty and nTMS response mask distance to the CST binary mask.

**Comparison with existing methods:**

We compare our approach (UncSeg) with the state-of-the-art TractSeg in terms of OC between the CST binary masks and nTMS response masks.

**Conclusions:**

In this study, we demonstrate that estimated uncertainty from UncSeg is a good measure of the agreement between the CST binary masks and nTMS response masks distance to the CST binary mask boundary.

## Highlights

• First study to evaluate nTMS as a validation technique for automated CST segmentation in tumor patients.
• CST segmentation shows significant overlap with positive nTMS motor responses.
• Estimated uncertainty effectively measures agreement between nTMS motor responses and distance to the segmented CST boundary.

## 1 Introduction

Deep learning-based (DL-based) approaches are currently the state-of-the-art for white matter (WM) tract segmentation (Wasserthal et al., 2018, Yeh et al., 2019, Wassermann et al., 2016, Siless et al., 2018, Garyfallidis et al., 2018, O’Donnell et al., 2017, Li et al., 2020, Zhang et al., 2020, Bert`o et al., 2021). DL-based methods are often trained using a supervised approach with tracts that were manually annotated on patients with healthy anatomy (Wasserthal et al., 2018). Although manual tract annotations can help during pre-operative planning, they are time-consuming and suffer from high inter-rater variability (Bert`o et al., 2021, Andreisek et al., 2010, De Schotten et al., 2011). Additionally, patients with abnormalities, such as brain tumors, can distort the tract anatomy, making accurate manual annotations challenging (Pujol et al., 2015). Furthermore, we recently presented a DL-based WM tract segmentation that uses uncertainty awareness for tract segmentation with accurate and reliable predicted probabilities so that clinicians can use it as a safety tool in pre-operative neurosurgical planning (Lucena et al., 2022) that we refer to as UncSeg.

Clinical validation of tractography is still an active research topic due to a lack of a clear gold standard for WM tract location (Pujol et al., 2015, Schilling et al., 2019). Direct cortical and subcortical stimulation (DCS) is used intra-operatively as the gold standard ground truth to determine the location of eloquent areas (Calabrese, 2016). However, this is an invasive technique that increases operation time and can bring a lot of fatigue to the patient in the awake setting, increases the length of surgery, require larger craniotomies for eloquent cortical exposure (Costabile et al., 2019, Krieg et al., 2017), and expose the patient to unnecessary risks – such as intra-operative seizures – if these tumors are proven not to be eloquent. Furthermore, resolving the differences in brain anatomy pre- and intra-operatively to evaluate pre-operative tractography is a complicated, and open research question (Gerard et al., 2021). For example, high-frequency techniques for subcortical mapping (such as the train-of-5 technique) provide a good estimation of the distance to the CST above 5 mA but are not reliable below this current (Nossek et al., 2011).

Navigated Transcranial Magnetic Stimulation (nTMS) is a well-established pre-operative brain mapping technique for motor function (Klomjai et al., 2015). In Neuro-Oncology, nTMS is widely used in pre-operative surgical planning given the recognized impact in the extent of resections, longer progression-free survival, and less post-operative motor deficits (Frey et al., 2014, Hendrix et al., 2021), and it is responsible for significant modifications in surgical approach in up to a third of the patients (Jung et al., 2019). Additionally, there are consistent reports from different groups that support high sensitivity and specificity in localizing the human primary motor cortex (M1) compared with DCS, even in patients with significant distortion of normal anatomy by pathology, such as brain tumors (Jung et al., 2019).

The corticospinal tract (CST) is one of the most important motor tracts in the human brain, being essential for fine motor activities of the hands and modulating walking pattern (Seo et al., 2012, Binkofski et al., 1996, Jang, 2009). nTMS was used as seeds for diffusion magnetic resonance images (dMRI) tractography of the CST (Weiss et al., 2015, Rosenstock et al., 2017) and to measure post-operative deficiency in tumors resection tractography (Muir et al., 2022, Raffa et al., 2018). nTMS positive motor responses act as reliable “silver” standard ground truth for motor mapping given its spatial resolution and documented correlation with DCS (gold standard ground truth) for motor mapping. To the best of our knowledge, no DL-based WM tract segmentation evaluation has used nTMS.

In this work, we evaluate the feasibility of using nTMS motor responses (positive and negative, inside or outside pre-central gyrus) as a method to assess the UncSeg segmentation algorithm for the CST in patients with diffuse astrocytic or oligodendroglial tumors. We focus our investigation on measuring concordance between CST segmentations and estimated uncertainty (Lucena et al., 2022) with nTMS motor response masks (“silver” standard ground truth for motor mapping). This investigation is a step towards a more informative non-invasive evaluation of brain mapping for pre-operative planning towards reducing the need to perform DCS for safe resection.

## 2 Methodology

### 2.1 Pipeline overview

We first describe the acquisition of dMRI and nTMS in patients (Section 2.2), steps taken for processing all data (Section 2.3). Then, we detail the DL tract segmentation approach (Section 2.4). Finally, we conduct the correspondence of dMRI and nTMS responses, where we assess the overlap between CST binary masks and nTMS responses and the correlation between estimated uncertainty and nTMS responses (Section 3).

### 2.2 Dataset description

*Inclusion Criteria:* Patients admitted at King’s College Hospital for surgery from March 2019 to March 2022 with presumptive preoperative diagnosis of motor eloquent - distance to M1 and/or CST *<* 10 *mm (*Raabe et al., 2014*)* - diffuse glioma in MRI and confirmed histological diagnosis of diffuse astrocytic or oligodendroglial tumor World Health Organization (WHO) grade 2–4; bi-hemispheric motor mapping for the functional area of the hand and pre-operative dMRI.

*Exclusion Criteria:* Incomplete clinical or pre-operative structural or mapping data. A total of 16 patients met the selection criteria (9 males, 7 females, 44*.*19(25*−*69) years old). Patient’s clinical and demographic characteristics are reported in Table 1. All patients were right-handed. The majority of the patients had left-sided lesions (75%) located in the frontal lobe (50%). No patient presented with focal motor deficits. The most common histological grading was WHO grade 3 (43*.*75%) and the most common histological type was oligodendroglioma (37*.*5%).

**Table 1:** Characteristics of the patient cohort. M denotes Male and F denotes Female. 16 patients were assessed at King’s College Hospital (9 males, 7 females, 44*.*19(25 *−* 69) years old).

Subjects	Age	Gender	Tumor location	Affected hemisphere	Tumor volume (cm^3^)	Diagnosis
1	34	M	Frontal	Left	98.1	Anaplastic Astrocytoma WHO Grade 3
2	51	F	Supplementary Motor Area	Right	45.3	Anaplastic Astrocytoma WHO Grade 3
3	47	M	Frontal	Left	129	Anaplastic Oligodendroglioma WHO Grade 3
4	40	F	Frontal	Left	207	Anaplastic Oligodendroglioma WHO Grade 3
5	25	M	Frontal	Left	10.1	Oligodendroglioma WHO Grade 2
6	36	F	Internal Capsule	Right	16.7	Glioblastoma WHO Grade 4
7	62	F	Subcentral	Right	6.67	Glioblastoma WHO Grade 4
8	59	F	Subcentral	Left	4	Glioblastoma WHO Grade 4
9	30	M	Supplementary Motor Area	Left	27.5	Astrocytoma WHO Grade 2
10	69	M	Fronto-Insular	Left	54.3	Glioblastoma WHO Grade 4
11	32	M	Temporo-Insular	Left	71.2	Anaplastic Oligodendroglioma WHO Grade 3
12	51	M	Temporo-Insular	Left	16.8	Glioblastoma WHO Grade 4
13	37	M	Temporo-Insular	Left	105.0	Anaplastic Oligodendroglioma WHO Grade 3
14	29	M	Temporo-Insular	Left	9.01	Astrocytoma WHO Grade 2
15	54	F	Frontal	Right	114.3	Astrocytoma WHO Grade 3
16	51	F	Frontal	Left	7.42	Oligodendroglioma WHO Grade 2

#### 2.2.1 Diffusion MRI (dMRI) acquisition

dMRI were acquired on a 1*.*5 T scanner with a field of view 243*×* 256*×* 176 mm. 64 gradient directions were acquired at b = 1500 s/mm^2^ and 6 images at b = 0 s/mm^2^.

#### 2.2.2 Navigated Transcranial Magnetic Stimulation (nTMS)

nTMS motor mapping was performed using the eXimia Navigated Brain Stimulation System (Nexstim, Helsinki, Finland), and a figure-of-8 coil (17 *×* 10 cm of diameter) was used as a pulse delivery tool, as published by (Jung et al., 2019, Lavrador et al., 2021). This system allows for precise and accurate localization of the electric field in the cortical tissue using a dynamic spherical model that is adjusted to the stimulation parameters. In practical terms, it is not the coil that is navigated by the electrical field itself which increases the precision of the mapping performed. A patient-specific registration is performed before the mapping procedure starts and an average error of less than 2 mm was ac- cepted before proceeding. The nTMS maps are exported in a DICOM format with a 1*mm*^3^ resolution. A standardized peeling depth was considered to minimize operator-dependent bias. In addition, each mapping had a quality check performed by the authors to ascertain the responses were located in the cortical-subcortical interface so the relation with the segmented corticospinal tract could be assessed.

Motor mapping for the functional areas of the hand was performed on both hemispheres so each patient act as their own control. Three upper limbs (UL) muscles are mapped for each functional area according to previously published protocols: *Abdutor Policis Brevis* (APB), 1st *Finger Flexor Digitus Interosseus* (FDI), and *Adutor Digitus Minimus* (ADM). The motor mapping is performed at 105% of the resting motor threshold and the criteria for positive motor evoked potentials were those established in the European Consensus for nTMS mapping using a figure-of-8 coil orientated 45 degrees to the central sulcus (Krieg et al., 2017). All nTMS responses obtained during the mapping with physiological latencies for the hand muscles (18 *−* 26 ms) were exported. They were divided into two groups: positive if amplitude above or equal to 50 µV or negative for amplitudes lower than this threshold.

**Fig. 1:**
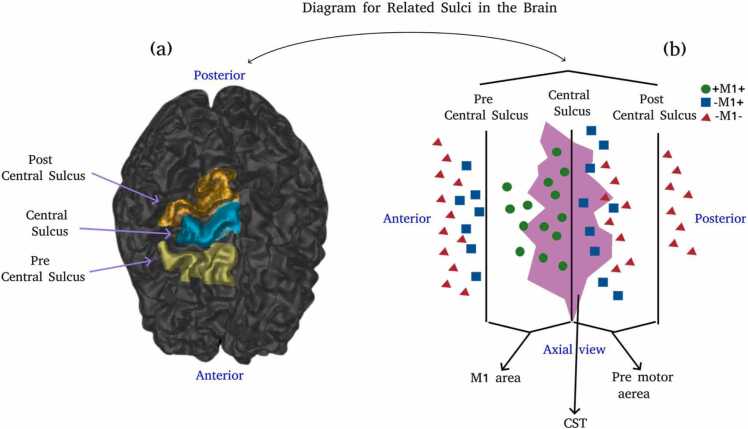
(a) Region used for nTMS stimulation. (b) The primary motor cortex is located anterior to the central sulcus and posterior to the pre-central sulcus (Nun˜ez et al., 2021). The CST originates mainly from the primary motor cortex and pre-motor area, while also receiving fibers from the somatosensory cortex, cingulate gyrus, and the parietal lobe (AbuHasan and Munakomi, 2022). In a subcortical division, the CST has most of its fibers in the central sulcus radiating to the primary motor cortex area (Seo et al., 2012, Yamada et al., 2007). Normally, the location of the nTMS responses follows the diagram where **-M1-** is furthest from the central sulcus, whereas **+M1+** and **-M1+** are the closest to the central sulcus.

Three nTMS mapping outputs are generated: **+M1+** (positive nTMS responses inside pre-central gyrus), **-M1+** (positive nTMS responses outside pre-central gyrus), and **-M1-** (negative nTMS responses outside pre-central gyrus). No map with a negative response within M1 is generated given the bias in its interpretation (most likely related to muscles not mapped during the nTMS mapping). The anatomical identification of M1 is performed based on anatomical landmarks on MRI (Voets et al., 2021) and the location of the motor hotspot - best motor response during nTMS mapping. Fig. 1 displays a diagram showing an example of anatomical identification of the primary motor cortex, and how the three masks described above were created. As a final processing step, nTMS responses underwent manual selection with regard to the effects of potential involuntary pre-activation and to eliminate false positive or false negative results. All nTMS responses export was performed at a chosen peeling depth of 25 mm from the scalp.

### 2.3 Data processing

#### 2.3.1 dMRI processing

All dMRI volumes are processed to reduce the effects of bias field, eddy current-induced distortion, motion, and susceptibility distortions (Tournier et al., 2019). As the tract segmentation method is trained on subjects in the MNI space, we rigidly register all dMRI data to the MNI space. Then, the dMRI signal intensities in the MNI space are transformed into spherical harmonic (SH) space without any model fitting for *l*_*max*_ = 4 to align data across different acquisitions without the need to fit a model to describe the tissue diffusivity/anisotropy. SH coefficients are then normalized by the b-zero shell using MRtrix (Tournier et al., 2019). Next, all SH values used as input for the tract segmentation model are clamped to the 5^*th*^ and 99^*th*^ percentile to remove outliers (Lucena et al., 2021).

#### 2.3.2 nTMS processing

nTMS motor responses are exported from eXimia Navigated Brain Stimulation System (See details in Section 2.2.2) with anatomical landmarks (*nasion*, *crus helicis* of each ear) used for co-registration with the T1w during nTMS mapping. Therefore, we first compute a binary mask corresponding to the intracranial region using FSL BET (Jenkinson et al., 2005) and MRtrix (Tournier et al., 2019) on the T1w. This binary mask is used to remove anatomical landmarks annotations from the nTMS responses. Secondly, we threshold the nTMS responses from the previous step with a value greater than zero to obtain nTMS response masks. Next, we downsampled the data to a voxel size equal to 1*mm*^3^ using NiftyReg (Modat et al., 2014) similar to (Weiss et al., 2015). Finally, WM segmentations derived using geodesic information flows (GIF) parcellation (Cardoso et al., 2015) were used to filter nTMS responses presented in WM locations to obtain the final nTMS response masks.

#### 2.3.3 nTMS alignment to tractography

We aligned the CST masks obtained by UncSeg in the MNI space to the nTMS T1w space to enable comparisons between the two (**+M1+**, **-M1+**, and **-M1-**), as follows: we rigidly register nTMS T1w (computed in Section 2.3.2) to the average dMRI of all b = 0 s/mm^2^ in the MNI space using FSL (Jenkinson et al., 2005). Then, we use the inverse transform from this registration to align the CST binary mask and uncertainty estimation from UncSeg to the nTMS T1w space.

### 2.4 Tract segmentation and uncertainty quantification

Segmentation of CST on dMRI is performed using UncSeg, a 3D CNN informative tract segmentation method previously published in (Lucena et al., 2022). UncSeg adopts 3D Unet as its backbone architecture and provides segmentation uncertainty quantification using test time dropout (TTD), test time augmentation (TTA), and a hybrid method that includes both TTD and TTA (Lucena et al., 2022). In this work, we used the Hybrid version of UncSeg to compute both epistemic (model’s parameters’ variability) and aleatoric (noise inherent in the data) uncertainties (Kendall and Gal, 2017).

As in (Lucena et al., 2022), we estimate epistemic uncertainty using test time dropout (Gal and Ghahramani, 2016), and aleatoric uncertainty using test time augmentation is implemented by performing multiple data augmentations to the input data at the inference stage in order to output multiple stochastic predictions (Wang et al., 2019). We compute *T* = 20 forward passes, where for each pass, we have a random data augmentation and a dropout rate *r* = 0*.*25. This gives a total of 20 predicted probabilities for each subject.

UncSeg is capable of directly segmenting WM tracts from SH coefficients. It outputs comparable tract segmentations to state-of-the-art methods, such as TractSeg (Wasserthal et al., 2018). Additionally, UncSeg provides segmentation uncertainty quantification which is a good measure of segmentation reliability (Lucena et al., 2022). Both UnSeg and TractSeg are trained on 105 healthy subjects from the Human Connectome Project (HCP) (Sotiropoulos et al., 2013), so their performance on patients with tumors have not been validated.

## 3 Experimental Design

### 3.1 Evaluation Metrics

We evaluate the quality of the CST binary mask based on its ability to accurately distinguish positive and negative nTMS responses using the Overlap coefficient (OC). OC is defined as the size of the union between ground-truth and prediction volumes over the size of the smaller set of the ground-truth and prediction volumes (Vijaymeena and Kavitha, 2016), in this case, nTMS is always the smaller set. OC ranges from 0, indicating the two sets are non-intersection, and 1, indicating the smaller set is fully enclosed by the larger set.

### 3.2 Experiments

We assess the relationship between the tract segmentation and nTMS response masks in the following scenarios: 1) overlap of the CST binary masks and nTMS response masks (*Overlap between CST segmentation and nTMS response masks*) and 2) how well the estimated uncertainty from nTMS response masks correlates to the distance to the CST binary mask (*Correlation between estimated uncertainty output from UncSeg and nTMS response masks distance to the CST binary mask*). The details of each experiment are described below.

#### 3.2.1 Overlap between CST segmentation and nTMS response masks

We assess how well the CST binary masks encompass nTMS response masks using OC described in Section 3.1. In this experiment, we use the pre-trained models provided by UncSeg to predict CST for all patients where the average predicted probability is threshold at 0*.*5 to generate a CST binary mask. We also compare the results of our method against the state-of-the-art DL-based model TractSeg. All the registration steps for dMRI and CST described in Section 2 were also used for TractSeg outputs. To assess the statistical significance of the OC, we used the Wilcoxon signed-rank (Haynes, 2013).

#### 3.2.2 Correlation between estimated uncertainty output from UncSeg and nTMS response masks distance to the CST binary mask

We assess how well the estimated uncertainty output from UncSeg correlates with nTMS response masks in terms of distance to the UncSeg CST binary masks. We compute a joint histogram with 30 bins between the estimated uncertainty output from UncSeg and the distance of the nTMS response masks to the CST binary masks from Experiment 1. Then, we compute the mean and standard deviation for each bin of the histogram. The correlation and the statistical significance of the joint histogram were measured using Spearman’s rank correlation coefficient (*ρ*) and its respective *p-value (*Sedgwick, 2014*)*. Spearmans’ correlation assesses monotonic relationships (whether linear or not). If there are no repeated data values, a perfect Spearmans’ correlation of 1 or *−* 1 indicates the variables are a perfect monotonic function of each other (Sedgwick, 2014).

## 4 Results

**Fig. 2:**
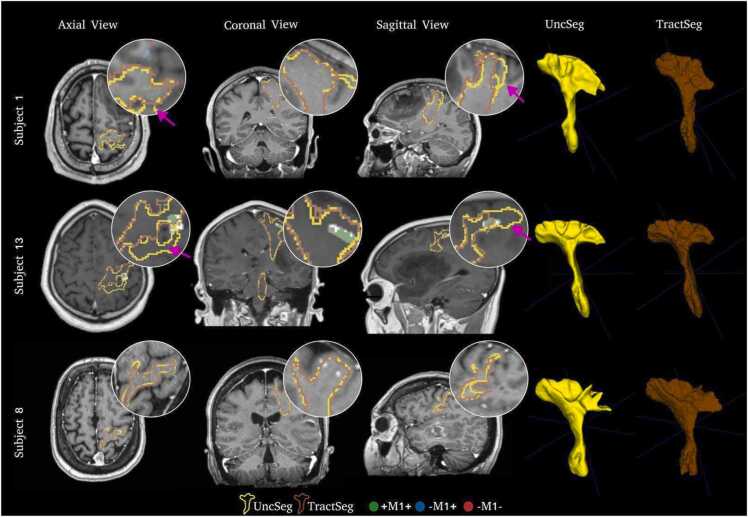
Correspondence between CST segmentations (yellow and brown) and nTMS response masks +M1+ (green dots), -M1+ (blue dots), -M1- (red dots) for Subjects 1, 8, and 13. 3D reconstructions of the CST segmentations are shown in yellow and brown for both UncSeg and TractSeg, respectively. White circles represent zoomed regions where there is an overlap between CST binary masks and nTMS response masks. Purple arrows show regions close to the post-central sulcus where UncSeg segments more than TractSeg.

**Fig. 3:**
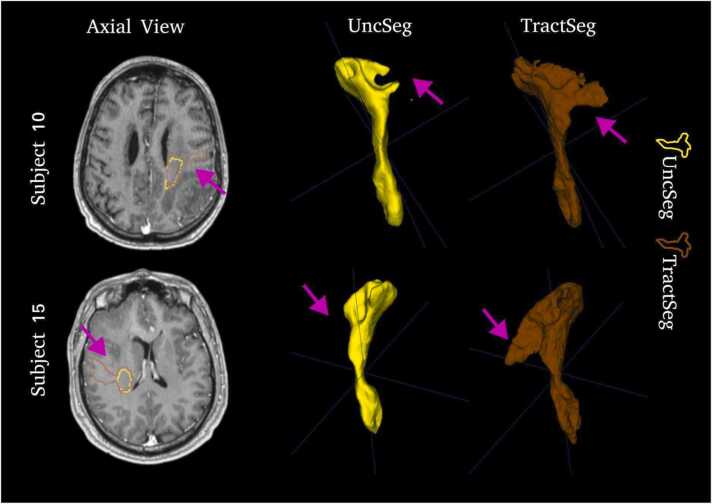
Comparison between CST binary masks output by UncSeg (yellow) and TractSeg (red) for Subjects 10 and 15. Purple arrows show regions where differences are most pronounced.

**Fig. 4:**
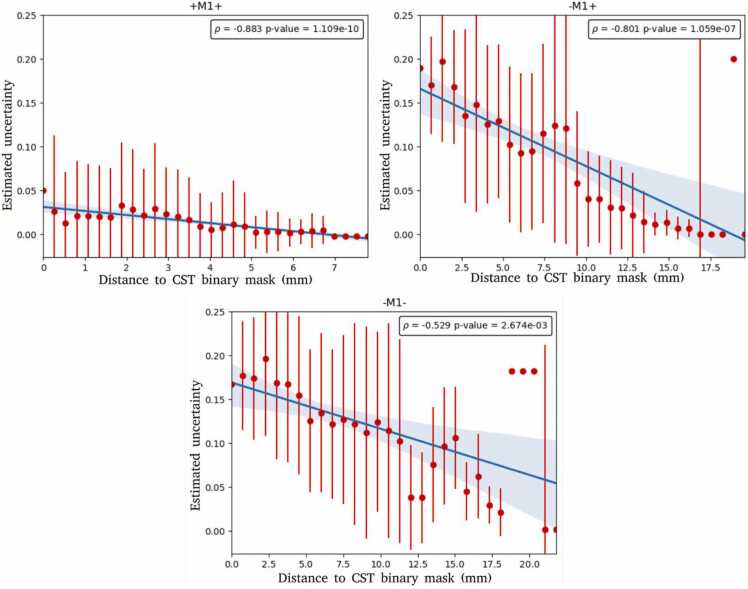
Joint histogram of UncSeg estimated uncertainty and nTMS response mask distance to the CST binary mask. A linear regression model is fit represented by the blue line where the red circles indicate the average uncertainty and the error bars indicate the standard deviation of the uncertainty estimation per bin. *ρ* indicates the strength of the correlation between UncSeg estimated uncertainty and nTMS response mask distance to the CST binary mask. *p-value* measures the statistical significance of the correlation.

OC is reported for all patients in Table 2, and qualitative segmentation results are shown in Fig. 2, Fig. 3. The correlation between the estimated uncertainty from UncSeg and the nTMS response mask distance to the UncSeg CST binary masks are presented in Fig. 4.

**Table 2:** Overlap coefficient (OC) between CST binary masks and nTMS response masks for all patients. The patients with the highest, average and lowest OC are indicated by bold text.

Subjects	Overlap coefficient						Dice overlap
	UncSeg			TractSeg			
	**+M1+**	**-M1+**	**-M1-**	**+M1+**	**-M1+**	**-M1-**	UncSeg vs. TractSeg
**1**	1.000	0.684	0.395	1.000	0.387	0.024	0.820
2	0.941	0.000	0.000	0.980	0.000	0.000	0.853
3	0.864	0.000	0.000	0.857	0.000	0.000	0.869
4	0.868	0.310	0.011	0.919	0.468	0.107	0.814
5	0.785	0.000	0.000	0.780	0.000	0.000	0.862
6	0.774	0.000	0.000	0.816	0.000	0.000	0.821
7	0.808	0.000	0.000	0.922	0.000	0.000	0.805
**8**	0.504	0.000	0.000	0.540	0.000	0.000	0.846
9	0.993	0.343	0.291	1.000	0.622	0.318	0.847
10	0.851	0.000	0.333	0.823	0.000	0.000	0.765
11	0.828	0.000	0.053	0.761	0.000	0.000	0.862
12	0.660	0.055	0.000	0.741	0.000	0.000	0.809
**13**	0.677	0.075	0.077	0.787	0.000	0.000	0.838
14	1.000	0.375	0.000	1.000	0.125	0.000	0.855
15	0.724	0.000	0.000	0.897	0.000	0.000	0.687
16	0.944	0.006	0.069	0.944	0.026	0.106	0.864

### 4.1 Overlap between tract segmentation and nTMS response masks

For UncSeg, CST binary masks have high OC for the **+M1+** (average OC = 0*.*83(0*.*14)), and low OC for the **-M1-** (0*.*00). This demonstrates a good concordance between the UncSeg CST binary masks and nTMS motor responses. Ideally, zero OC is expected for **-M1-**, as these are points where no motor response was observed for nTMS. Nonetheless, when these responses are located close to the central sulcus, where the core of the CST is located, the CST binary mask may encompass a few **-M1-** (i.e. Subject **13**, Fig. 2). However, the pattern OC(**+M1+**) *>* OC(**-M1+**) *>* OC(**-M1-**) is observed in all patients as expected.

Fig. 2 shows segmentations for subjects from Table 2 with the highest, average, and lowest OC. UncSeg generates good CST segmentations (3D reconstructions, Fig. 2). However, when nTMS responses are located close to the WM and gray matter (GM) boundary (regions with more uncertainty), a low OC for the **+M1+** is observed (Subject **8**).

Both UncSeg and TractSeg have comparable CST binary masks (Dice overlap, Table 2) with small variations, for example on subjects **10** and **15** where TractSeg has segmented more of the pre-central part of the CST (Fig. 3). However, these differences do not influence the OC observed for **+M1+** (Table 2).

For the OC(**+M1+**), UncSeg demonstrates comparable performance to TractSeg, where average OC across patients = 0*.*83(0*.*14) for UncSeg and = 0*.*86(0*.*12) for TractSeg, with a *p-value* = 0*.*04. Although this difference is statistically significant, the difference between the methods is small *≈* 0*.*03. This can be explained due to more “blocky” fanning output by TractSeg that includes more responses when compared to UncSeg which has a smoother CST segmentation (Fig. 2). The “blocky” fanning is most likely caused by TractSeg using a 2D tri-planar approach where 3D context information is not properly taking into account whereas UncSeg is a fully 3D segmentation approach. No statistical significance differences are found for OC(**-M1+**) and OC(**-M1-**), *p-value* = 0*.*60 and *p-value* = 0*.*25, respectively.

### 4.2 Correlation between estimated uncertainty output from UncSeg and nTMS response masks distance to the CST binary mask

Fig. 4 displays estimated uncertainty from UncSeg versus nTMS response mask distance to the CST binary mask. A strong negative correlation between estimated uncertainty and nTMS response mask distance to the CST binary mask is an indication of how reliably UncSeg estimated uncertainty corresponds to uncertainty in the nTMS responses, where low segmentation uncertainty should indicate that the nTMS response is located far from the CST boundary.

For all nTMS response masks, a statistically significant strong negative correlation is found between estimated uncertainty and nTMS response mask distance to the CST binary mask (Fig. 4). Low variability in the estimated uncertainty is found for **+M1+** while a higher variability in the estimated uncertainty is found for both **-M1+** and **-M1-** as expected due to CST anatomy being located mostly in the primary motor cortex (See Fig. 1). High variability in the estimated uncertainty is observed in regions located *<* 10 mm away from the CST binary mask, which is relatively close to the CST boundary. Variability in the estimated uncertainty decreases further.

from the CST binary mask boundary. This demonstrates the estimated uncertainty is correlated with regions of high uncertainty between the UncSeg CST binary masks and nTMS response masks and may help identify false positive locations in the segmentation.

For the **+M1+** , the correlation has a good fit with no outliers, demonstrating high reliability of UncSeg estimated uncertainty related to the nTMS responses. For the **-M1+** and **-M1-**, some outliers are found for regions located *>* 12*.*5 mm away from the tract. One possible explanation is UncSeg tends to segment as belonging to the CST regions in the post-central sulcus, which are regions that include more **-M1-** and are more likely to have higher variability in the uncertainty estimation. Finally, the shape of the tract may be distorted when close to tumor regions which can impact the outputs of UncSeg.

## 5 Discussion

We assessed how well a DL-based CST segmentation (UncSeg) relates to nTMS motor responses for patients with motor eloquent diffuse gliomas. We showed quantitatively (Table 2) that CST binary masks output from UncSeg have high OC with positive nTMS response masks within the primary motor cortex and the pattern of OC(**+M1+**) *>* OC(**-M1+**) *>* OC(**-M1-**) was observed in all patients, as expected. UncSeg also outputs comparable CST segmentations to TractSeg, one of the current state-of-the-art methods for tract segmentation (Fig. 2). A significant strong negative correlation was observed between the estimated uncertainty computed by UncSeg and the nTMS response masks distance to CST binary masks, which indicates uncertainty output by UncSeg is a good measure of agreement between the CST binary masks and nTMS response masks (Fig. 4).

Although UncSeg and TractSeg have very similar performance, UncSeg segments more CST regions close to the post-central sulcus compared to TractSeg (Purple arrows, Fig. 2). However, for **-M1+** , prior knowledge of CST anatomy (Penfield and Boldrey, 1937) suggests responses posterior to the post-central sul- cus regions are more likely to be true positives, and responses anterior to the pre-central sulcus are more likely to be false positives. Because there is a known bias in the portion of true positives and false positives in **-M1+** based on anatomical location, OC may appear higher when compared to TractSeg, suggesting UncSeg has more concordance to the true location of the CST than TractSeg. However, it is not possible within this given study to accurately measure true correspondence to CST location as nTMS is only a “silver standard” ground truth. UncSeg’s main advantage over TractSeg is its ability to provide uncertainty awareness for tract segmentation. It offers accurate and reliable predicted probabilities, allowing clinicians to use it as a safety tool in preoperative neurosurgical planning (Lucena et al., 2022), and in this work, we show a strong negative correlation observed between the estimated uncertainty and the nTMS response masks distance to CST binary masks. Experienced clinicians can benefit from the correlations computed using un- certainty maps, as they provide additional information. On the other hand, less experienced clinicians can make more use of uncertainty maps to identify abnormal regions and unexpected white matter anatomy.

Our study was subject to many sources of variability. First, tumors can distort anatomy and shift the location of the tracts, especially when they have large volumes and significant mass effect and/or edema, making it hard to have a good delineation of the CST (Pujol et al., 2015). Since UncSeg is only trained on healthy subjects, we expect it to have a poorer performance on the subjects in this dataset compared to the results reported in (Lucena et al., 2022). Secondly, all patients had nTMS motor responses exported with 25 mm peeling depth from the scalp, which does not consider the inter-subject variability of scalp and skull thickness and can result in nTMS responses far from WM/GM boundary. In this context, we would expect an increase in segmentation uncertainty for areas with high variability of the anatomy and high certainty in areas with high concordance across subjects. Thirdly, we set a threshold of 0*.*5 to output CST binary masks. The segmentation of the CST was independent of the nTMS responses. However, the nTMS responses were exported with 1*mm*^3^ interpolated resolution and therefore the overlap coefficient may be affected in the edge of the segmentation of the CST. Additionally, distortions in anatomy due to the presence of tumors may result in under-segmentations, and fine-tuning this threshold may improve segmentation accuracy.

Although UncSeg was trained on anatomically healthy subjects, we observe good reconstructions for the CST, with high concordance in terms of uncertainty, when compared to the “silver” standard nTMS motor responses. However, a key limitation in our analysis is UncSeg cannot take into account WM distortions not presented in the training set. Therefore, uncertainty from patients where tumors considerably distort the CST anatomy will be computed based on in-distribution healthy subject data, and further characterization is needed to flag out-of-distribution (OoD) data for patients with tumors not adequately captured by the current model limit models from failing silently (Graham et al., 2022, Linmans et al., 2023).

The clinical validation of tractography remains an active area of research due to the absence of a clear gold standard for determining white matter tract location (Pujol et al., 2015, Schilling et al., 2019). Additionally, tumors can shift or distort tracts and fibers can go through the tumor which can impair the quality of the ground truth annotation (Pujol et al., 2015, Essayed et al., 2017), making segmentation comparisons challenging. In a clinical setting, electrical activity accessed through either DCS or nTMS is used, as these methods can identify eloquent cortex areas. Therefore, despite the limitations discussed above, this is the first work focusing on assessing the feasibility of using nTMS motor responses as a means to evaluate DL-based tract segmentation. We demonstrate OC has a consistent and expected relationship between nTMS responses and CST binary masks. Furthermore, this trend is consistently observed for two different tract segmentation methods (UncSeg and TractSeg). Finally, the correlation between uncertainty estimation and the distance of the nTMS response masks to the CST binary masks showed a strong correlation indicating the estimated uncertainty is a good measure of model reliability. Our investigation is a step toward increasing the reliability and reproducibility of pre-operative brain mapping for eloquent areas on clinically acquired data in patients with tumors.

## 6 Conclusions

In this work, we assessed the feasibility of using navigated transcranial magnetic stimulation (nTMS) motor responses as a method to assess an informative deep learning (DL)-based tract segmentation method (UncSeg) for the corticospinal tract (CST). We demonstrate that 1) CST binary masks output from UncSeg have a high overlap coefficient with navigated transcranial magnetic stimulation (nTMS) response masks and 2) estimated uncertainty is strongly correlated to nTMS response masks distance to the CST binary mask. In this study, we used UncSeg trained on healthy subjects to assess concordance between estimated CST segmentation uncertainty and nTMS responses. These analyses do not take into out-of-distribution (OoD) distortions absent in the training set. Therefore, future validation taking into consideration OoD uncertainty requires quantification. The use of OoD uncertainty may more effectively identify cases where, for example, tumor sizes/ malignancy distort anatomy in a way unknown to the model.

## Ethics Statement

All data were evaluated retrospectively. All studies involving human participants were in accordance with the ethical standards of the institutional and/or national research committee and with the 1964 Helsinki declaration and its later amendments or comparable ethical standards.

## CRediT authorship contribution statement

**Vergani Francesco:** Resources. **Borges Pedro:** Writing – review & editing. **Sparks Rachel:** Writing – review & editing, Writing – original draft, Supervision, Conceptualization. **Granados Alejandro:** Writing – review & editing. **Ourselin Sebastien:** Writing – review & editing, Supervision. **Ashkan Keyoumars:** Writing – review & editing, Supervision. **Lucena Oeslle:** Writing – review & editing, Writing – original draft, Visualization, Validation, Software, Resources, Methodology, Investigation, Formal analysis, Data curation, Conceptualization. **Irzan Hassna:** Writing – review & editing. **Lavrador Jose Pedro:** Writing – original draft, Conceptualization. **Semedo Carla:** Writing – review & editing.

## Declaration of Competing Interest

We wish to confirm that there are no known conflicts of interest associated with this publication and there has been no significant financial support for this work that could have influenced its Outcome.

We confirm that the manuscript has been read and approved by all named authors and that there are no other persons who satisfied the criteria for authorship but are not listed. We further confirm that the order of authors listed in the manuscript has been approved by all of us.

We confirm that we have given due consideration to the protection of intellectual property associated with this work and that there are no impediments to publication, including the timing of publication, with respect to intellectual property. In so doing we confirm that we have followed the regulations of our institutions concerning intellectual property.

We understand that the Corresponding Author is the sole contact for the Editorial process (including Editorial Manager and direct communications with the office). He/she is responsible for communicating with the other authors about progress, submissions of revisions, and final approval of proofs. We confirm that we have provided a current, correct email address that is accessible by the Corresponding Author and which has been configured to accept emails from oeslle.lucena@kcl.ac.uk.

## Data Availability

The authors do not have permission to share data.
